# Increased heart fibrosis and acute infection in a murine Chagas disease model associated with organophosphorus pesticide metabolite exposure

**DOI:** 10.1038/s41598-019-54218-7

**Published:** 2019-11-26

**Authors:** Dunia Margarita Medina-Buelvas, Elizabet Estrada-Muñiz, Miriam Rodríguez-Sosa, Mineko Shibayama, Libia Vega

**Affiliations:** 1 Department of Toxicology, Centro de Investigación y de Estudios Avanzados del Instituto Politécnico Nacional, Av. Instituto Politécnico Nacional 2508, San Pedro Zacatenco, CP 07360 Gustavo A. Madero, Ciudad de México México; 2 Biomedicine Unit, Facultad de Estudios Superiores Iztacala, Universidad Nacional Autónoma de México (UNAM), Avenida de los Barrios 1, Los Reyes Iztacala, CP 54090 Tlalnepantla, Estado de México México; 3 Department of Infectomics and Molecular Pathogenesis, Centro de Investigación y de Estudios Avanzados del Instituto Politécnico Nacional, Av. Instituto Politécnico Nacional 2508, San Pedro Zacatenco, CP 07360 Gustavo A. Madero, Ciudad de México México

**Keywords:** Parasitic infection, Innate immunity, Cardiology, Toxicology

## Abstract

Some reports suggest that exposure to organophosphorus (OP) pesticides increases the incidence of infections. Ethylated dialkylphosphates (EtDAPs) are metabolites of OP pesticides widely distributed with immunomodulatory potential. Chagas disease is produced by *Trypanosoma cruzi* parasites, and resolution of this infection requires the activation of inflammatory macrophages (MΦ), which results in cardiac fibrosis. Some reports indicate that EtDAPs increase the amount of the anti-inflammatory alternatively activated MΦ (M2; CD206^+^F4/80^+^). Therefore, we analyzed the course of *T. cruzi* infection, MΦ profiles from peritoneal exudate cells (PECs), inflammatory cell infiltration and fibrosis in the heart of BALB/c mice exposed to diethyldithiophosphate (DEDTP), diethylthiophosphate (DETP) or diethylphosphate (DEP, 0.01 g/kg), common DAPs produced by OP pesticides, 24 h before infection with *T. cruzi*. We found that DEDTP increased the parasite burden in blood by 99% at the peak of the infection and enhanced the myocardial damage due to an increase in infiltrated inflammatory cells (induced by DEDTP or DETP) and fibrosis (induced by EtDAPs). In the PECs, exposure to EtDAPs increased the proportion of the MΦ subpopulations of M2a, M2b and M2d, which are associated with tissue repair. These results indicate that exposure to EtDAPs can exacerbate the acute phase of a parasitic infection and increase the long-term damage to the heart.

## Introduction

Chagas disease (ChD), or American trypanosomiasis, is caused by the infection of *Trypanosoma cruzi* protozoa, primarily in MΦ, which the parasite uses to propagate its infection^1^. ChD is a public health problem and is considered a neglected tropical disease (NTD) that affects 6 to 7 million persons worldwide^2^, with 1.1 million individuals infected in Mexico and 29.5 million at risk of infection^3^. However, a previous meta-analysis of epidemiological surveys from 2006 to 2017 determined that the infection has been underestimated and suggest that 4.06 million cases are active, with an estimated annual cost *per* patient between $6,700 and $11,838 USD, increasing the annual cost for medical care^3^.

Chagas cardiomyopathy (CC) is one of the chronic manifestations of *T. cruzi* infection; approximately 30% of chronically infected individuals develop CC 20–30 years after the initial parasitic infection^4^. In Latin America, ChD is responsible for as many as 41% of heart failure (HF) cases in endemic areas; the pathogenesis of CC is not completely understood, but it has been suggested that the development of myocardial damage is due to parasitic invasion and the severe immune inflammatory response that follows, leading to fibrosis and cellular hypertrophy. These alterations can induce disturbances in cardiac rhythms and induce myocardial abnormalities, aneurysms and thromboembolism phenomena, which lead to progressive HF and sudden cardiac death^5^.

Parasite-infected individuals are continually interacting with pesticides in their environment that are known to modify some immune responses, changing the outcome of the infection and the development of the disease. A previous report presented a theoretical model based on a system of nonlinear ordinary differential equations (ODE) to predict the possible interactions and the relationships between toxicant exposure, immune response and parasite infection of an organism after sublethal exposure. The results suggested that sublethal toxicant exposure intensifies the infection levels because the parasite density increases quickly within the host as a result of exposure to immunotoxicants^6^.

Organophosphorus (OP) pesticides and other OP compounds constitute an important group of pollutants produced in large quantities and are commonly used today as additives for lubricants, plasticizers, flame retardants^7,8^ and common insecticides. In particular, insecticides are widely used to eradicate deadly vector-borne illnesses and agricultural/urban pests in several regions around the world^9,10^. Some reports indicate that, in 2006, approximately 91,000 tons of OP pesticides were applied worldwide, an amount that has increased rapidly, reaching 680,000 tons between 2011 and 2015^11,12^. In 2017, the Food and Agriculture Organization (FAO) reported that China was the principal consumer of pesticides (1,807,000 tons of active ingredients) and that Mexico ranked fifth in pesticide use (98,814 tons of active ingredients)^9^. Because of the current use and widespread distribution of OP compounds, it is not surprising that the continuous exposure to pesticides contributes to a wide variety of long-term adverse effects on human health^13^.

Several organisms can metabolize OP compounds (e.g., humans, plants, and bacteria) that are degraded easily by environmental factors (e.g., light, pH, and temperature), generating byproducts such as methylated or ethylated dialkylphosphates (DAPs). These ethylated DAPs (EtDAPs) are widely dispersed in the environment (e.g., food, water, soil, and air), increasing exposure to these contaminants globally in the general population, specifically in occupationally exposed populations^14^.

Diethyldithiophosphate (DEDTP, CAS no. 298-06-6), diethylthiophosphate (DETP, CAS no. 2465-65-8) and diethylphosphate (DEP, CAS no. 598-02-7), known EtDAPs, are common detoxification/excretion metabolites used as biomarkers of pesticide exposure and are generally considered harmless due to their high stability and persistence in different environments^14,15^.

Some epidemiological studies report that exposure to OP pesticides may increase the rate and risk of infections in rural populations^16^, exacerbating cutaneous leishmaniasis and inducing a low response to treatment in farmers exposed to chlorpyrifos, an OP pesticide that generates DETP and DEP^17^. *In vivo* studies showed that these effects on the immune response may be due to a decrease in the levels of plasma nitric oxide (NO) and interferon (IFN)-γ and to the increase in the activity of arginase, which explains the inability of macrophages (MΦ) to control the parasites or induce an adequate immune response against them^18^.

In this regard, some reports indicate that EtDAPs produce cytostaticity in T CD8^+^ (cytotoxicity-inducing) lymphocytes^19^ and unresponsiveness in T CD4^+^ (helper) lymphocytes^20^ using *in vitro* models, suggesting that DEDTP has the potential to induce immunotoxicity. In addition, an *in vivo* study showed that exposure to DEDTP induced immunotoxicity in T and natural killer (NK) cells, but the most relevant immunomodulatory effect of DEDTP exposure was observed in MΦ, mainly M2-type cells, the number and activation of which were dramatically increased^21^.

MΦ have great functional diversity^22,23^. MΦ can polarize towards a classic phenotype with inflammatory and microbicidal properties (M1) or can acquire an alternative phenotype that is involved in the resolution of inflammation, reestablishment of homeostasis, and tissue repair; known as M2, this alternative phenotype is further classified into four different subtypes (M2a, b, c and d)^24^. Experimental evidence suggests that OP compounds modify the MΦ response, inducing a suppressor phenotype *in vitro*^25–28^. This alteration of MΦ can affect other biological responses, such as the restoration of normal architecture of tissues during an infection. Thus, a dysregulated wound healing process can lead to the development of fibrosis (an excessive accumulation of fibrillar collagen-rich extracellular matrix, ECM) that replaces functional tissue. Extensive fibrosis can lead to organ failure or can even cause death when affecting organs such as the heart^29^.

In this study, we evaluated how exposure to EtDAPs affects the course of short- and long-term development of *T. cruzi* infection (parasite burden, cardiac inflammation and fibrosis) in mice as it relates to the alteration of the M1/M2 (M2a, M2b, M2c or M2d) subset distribution. The results suggested that exposure to EtDAP influences the immune response to *T. cruzi* infection by altering the polarization of the MΦ phenotype.

## Results

### Exposure to EtDAPs modified the number of parasites in the peripheral blood during acute murine ChD

As a first step, we determined whether the EtDAPs induced a direct toxicity-inducing response to a low-virulence *T. cruzi* Ninoa strain (classified as *T. cruzi* I); we exposed the parasites to concentrations ranging from 1 to 25 µM of DEDTP, DETP or DEP for 24 h and performed the MTT assay to evaluate the viability of the parasites. The results showed that exposure to EtDAPs did not affect the viability of the parasites by more than 20% at any concentration tested (Supplementary Fig. S1A). We also evaluated the directly induced toxicity of EtDAPs on the PECs and observed that exposure to the EtDAPs did not reduce the viability of the PECs at any of the concentrations used (Supplementary Fig. S1B). From these results, we considered the dose of 1 µM, equivalent to 0.01 g/kg body weight, as a noncytotoxic dose to evaluate the effect of EtDAP exposure on the development of a parasitic infection *in vivo*. This dose is within the range of DAPs found in biological samples of humans nonoccupationally exposed to OP pesticides^15,30,31^.

**Figure 1:**
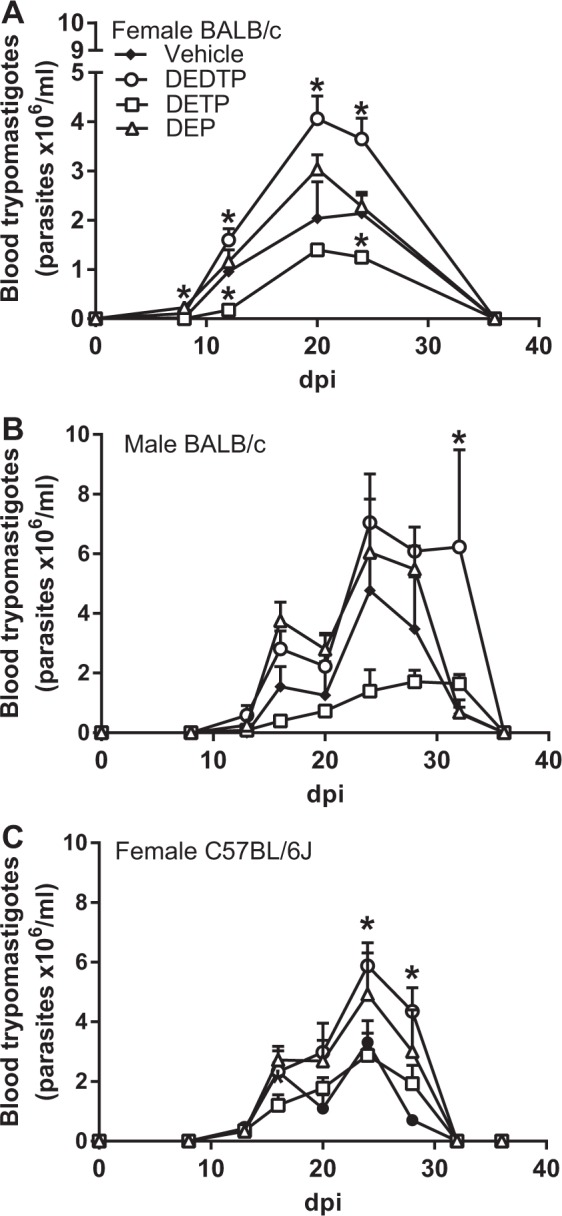
Effect of EtDAPs on *T. cruzi* trypomastigote parasite burden in peripheral blood of female BALB/c (**A**), male BALB/c (**B**) or female C57BL/6J (**C**) mice. Mice were i.p. injected with 0.01 g/kg body weight of DEDTP, DETP or DEP in corn oil (50 µl) and i.p. infected with 7.5 × 10^3^ bloodstream trypomastigotes of *T. cruzi* 24 h after the exposure. Parasitemia was evaluated at 8, 12, 20, 24 and 36 dpi. Data represent the mean ± SE (n = 8). *p ≤ 0.05, one-way ANOVA *post hoc* Bonferroni test of samples *vs*. vehicle.

We first evaluated the effect of exposure to a single dose of 0.01 g/kg body weight (i.p. administered) EtDAPs 24 h before the infection (i.p.) with 7.5 × 10^3^ blood trypomastigotes in a susceptible mouse strain with a Th2 genetic background (female BALB/c mice) and compared the results with those from infected nonexposed animals (vehicle). We found that the data on the animals exposed to DEDTP, DETP and DEP and infected with *T. cruzi* Ninoa generated a similar infection curve, as determined by the parasite counts in the blood over time. The results showed a rapid and continuous increase in parasitemia with similar behaviors in the acute phase (8 days), at peak parasitemia (day 20) and during parasite clearance (between days 24 and 36), while the vehicle group presented peak parasitemia at day 24 (Fig. 1A, filled figure). The mice exposed to DEDTP showed a significantly higher level of blood parasitemia from day 12 to 24 (from 67 to 71%) with peak parasitemia at day 20, which indicated an increase of 99% in the parasite burden than was observed in the nonexposed mice (Fig. 1A, empty circles). The mice exposed to DEP showed a significant increase in the parasite burden at day 8 (50%). Nevertheless, no significant differences were observed over time in the parasitemia curves of the DEP exposed mice and the nonexposed animals (Fig. 1A, empty triangles). The mice exposed to DETP showed a slower parasite replication rate and a decrease in parasite burden in the blood at 12 (81%) and day 24 (42%), than shown by the nonexposed animals (Fig. 1A, empty squares).

To examine whether sex (female *vs*. male) or the genetic immunological background (Th2 *vs*. Th1) of the mice played a relevant role in the effect of the EtDAP exposure on the response to *T. cruzi* infection, we evaluated the parasitemia curve in male BALB/c and female C57BL/6J mice. We observed that male mice exhibited a rapid and continuous increase in parasitemia starting at the acute phase at day 14, peak parasitemia at day 24 and parasite clearance approaching day 36 (Fig. 1B, filled figure). As with female BALB/c mice, we observed that male BALB/c mice exposed to DEDTP 24 h prior to infection had significantly increased parasitemia in blood at day 32 (873%) compared with the level in the blood of the nonexposed male mice, although the peak of the parasitemia was observed on the same day for both the exposed and nonexposed animals (Fig. 1B, empty circle). The parasite count in the blood indicated that the BALB/c male mice previously exposed to DEDTP or DEP for 24 h had a higher parasite burden (1.07-fold and 2-fold, respectively) than the female mice (Fig. 1B, empty circles and triangles, respectively). With the BALB/c male mice, we also observed that exposure to DETP induced a reduction in the parasite burden, as also observed in the female mice. Although female BALB/c mice showed a more homogeneous response than males, we decided to continue our experiments with the female mice.

Furthermore, we evaluated the effect of EtDAP exposure in C57BL/6J female mice, a strain with a strong Th1 genetic background that is less susceptible to infection due to their high basal proinflammatory profile^32,33^. In these mice, we observed that exposure to DEDTP and DEP increased blood parasitemia at the peak of infection between 24 days (19%) and 28 days (45%), while exposure to DETP reduced the number of blood parasites in the early stages of infection at day 13 (50%), and this effect did not differ from that of the nonexposed animals in the subsequent evaluation at later times (Fig. 1C). Despite their different responses, all animals resolved the acute infection by day 32. When we compared the effect of EtDAP exposure on the responses in the Th2 (BALB/c) and Th1 (C57BL/6J) mice, we observed that the acute phase of parasitemia was resolved earlier in the C57BL/6J female mice than it was in the BALB/c female mice, but the trend of the infection curve in EtDAP-exposed animals was similar for both mouse strains. As we noted in earlier experiments, the alterations in the course of the infection were more evident and significant in the female mice, although we did not observe clear or significant changes in males of the same strain because the results from experiments with them were more variable. Thus, we decided to use only BALB/c female mice for the other experiments.

### EtDAPs promoted heart inflammation during chronic ChD

**Figure 2:**
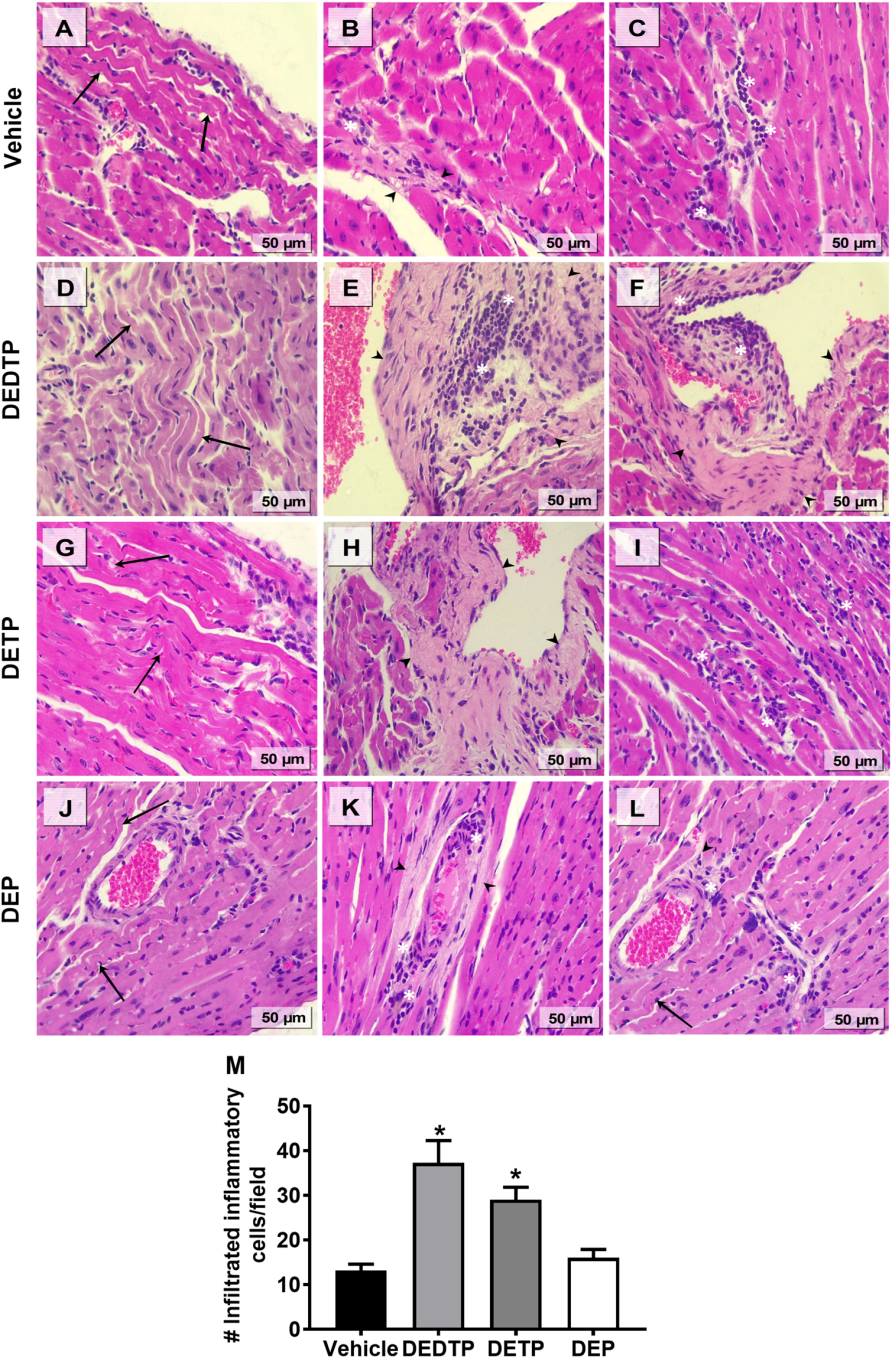
Effect of EtDAPs on the heart of a murine model of chronic ChD. Representative images from hematoxylin and eosin (H&E) staining of cardiac fiber destruction (black arrows), collagen deposition (closed arrowhead) and inflammatory reaction (white asterisk) on heart sections (5 µm) from female BALB/c mice after injection of corn oil vehicle (**A**–**C**) or 0.01 g/kg body weight of DEDTP (**D**–**F**), DETP (**G**–**I**) or DETP (**J**–**L**) and infection with 7.5 × 10^3^ bloodstream *T. cruzi* trypomastigotes after 180 days. Bar = 50 μm. Quantification of inflammatory cells (**M**). Data represent the mean ± SE (n = 5) evaluating 20 fields per sample. *p ≤ 0.05, one-way ANOVA *post hoc* Bonferroni tests of samples *vs*. vehicle.

The heart is one of the most important organs affected by chronic ChD, and HF is associated with lethality in individuals exposed to OP pesticides. To determine whether the initial exposure to EtDAPs during the acute phase of ChD could change the outcome of the disease in the chronic phase, we performed morphological and histological examinations of cardiac tissue of the mice exposed to DEDTP, DETP or DEP (0.01 g/kg, i.p.) and infected with *T. cruzi*. At 180 dpi, the histological analysis of the heart from the nonexposed animals demonstrated minor damage that had been induced by chronic *T. cruzi* infection, as characterized by minimal cardiac fiber destruction (Fig. 2A), presenting as areas with slight collagen deposition (Fig. 2B) and few *foci* with immune cell infiltration (Fig. 2C). In contrast, the heart of the mice exposed to DEDTP or DETP showed a higher extent of cardiac fiber destruction (Fig. 2D,G), elevated collagen deposition (Fig. 2E,H) and a higher number of inflammatory cells (Fig. 2F,I) compared with the number in the nonexposed animals. In addition, the cardiac damage observed in the heart of mice exposed to DEP was slightly higher than that observed in the nonexposed animals, but the differences in the number of inflammatory cells *per* field for the exposed and the nonexposed animals were not significant (p < 0.05) (Fig. 2M); the number of inflammatory cells is one characteristic of myocarditis. Our results also showed that exposure to DEDTP or DETP significantly increased the number of inflammatory cells infiltrated in the heart (183 and 121%, respectively).

Although we did not observe a significant increase in heart tissue damage in the DEP-exposed mice, we observed an abnormal decrease in the weights of their hearts (14%), spleens (16%) and kidneys (13%), indicating a different systemic effect of DEP. However, although exposure to DEDTP and DETP produced high levels of tissue damage in the heart, it did not significantly modify the weight of any of the organs studied (Table 1). It is important to establish that, despite the tissue damage in the heart, no amastigote nests were observed in any group 180 dpi, including the mice with a higher infection rate (2 × 10^4^ bloodstream *T. cruzi* trypomastigotes) (Supplementary Fig. S2E–G).

**Table 1:** Relative organ weights of female BALB/c mice after 180 dpi of EtDAPs exposure and inoculation with 7 × 10^3^ bloodstream trypomastigotes of *T. cruzi* Ninoa.

Relative weight (%)	Hearts	Spleens	Livers	Lungs	Kidneys
**Vehicle**	0.50 ± 0.02	0.63 ± 0.02	5.74 ± 0.19	0.93 ± 0.04	0.71 ± 0.01
**DEDTP**	0.50 ± 0.05	0.60 ± 0.03	5.95 ± 0.16	0.98 ± 0.10	0.68 ± 0.03
**DETP**	0.48 ± 0.07	0.65 ± 0.03	5.94 ± 0.15	0.98 ± 0.11	0.72 ± 0.03
**DEP**	0.43 ± 0.02*	0.53 ± 0.04*	5.74 ± 0.19	0.74 ± 0.21	0.62 ± 0.02*

*p ≤ 0.05, Student’s t-test of samples *vs*. vehicle, n = 5, mean ± SE.

### EtDAPs increased heart fibrosis damage during chronic ChD

**Figure 3:**
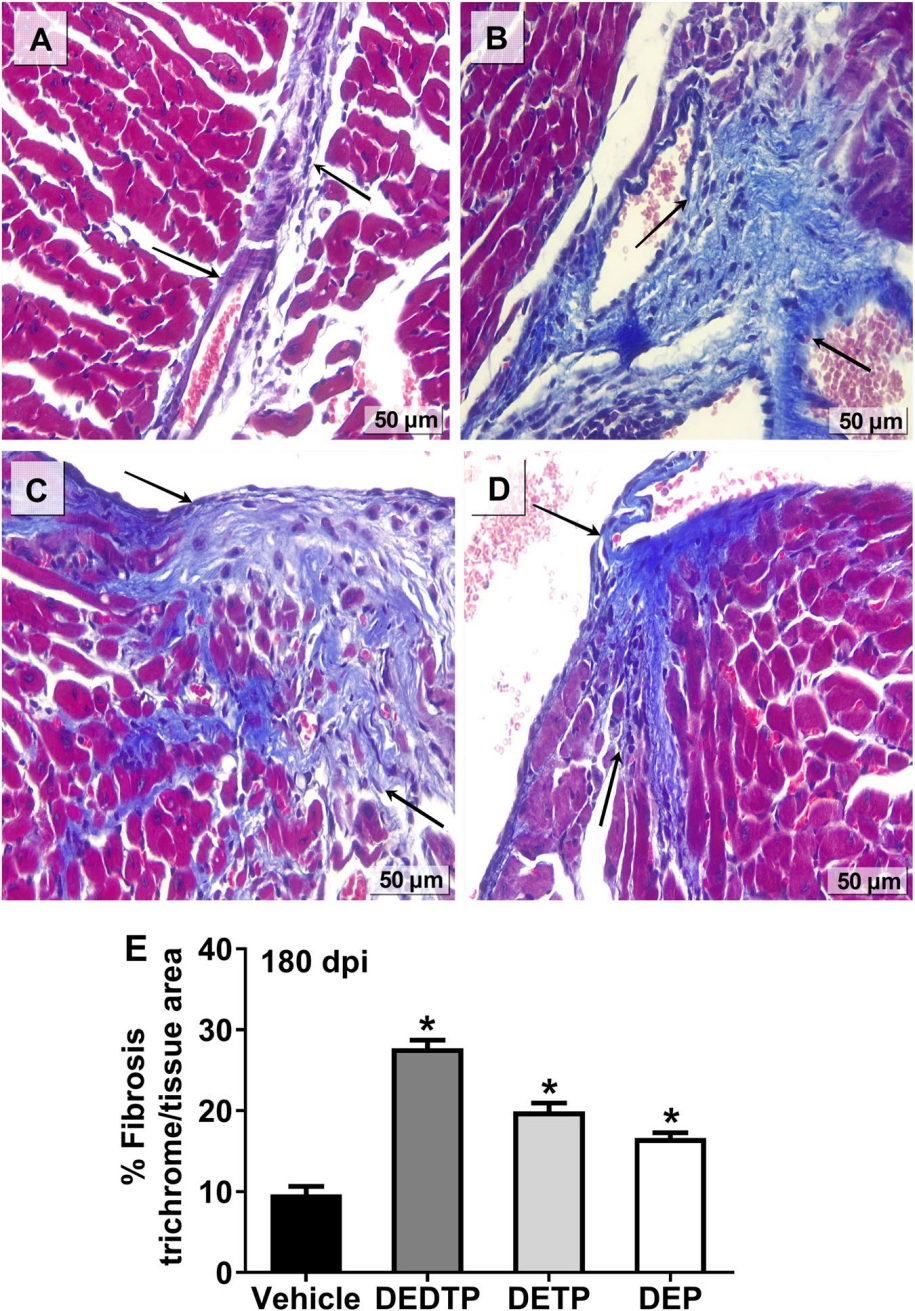
Effect of EtDAP exposure on the fibrotic response to myocardial damage induced by *T. cruzi* infection. Female BALB/c mice after 180 days of injection with corn oil vehicle (**A**) DEDTP (**B**) DETP (**C**) or DEP (**D**) at 0.01 g/kg body weight and i.p. infection with 7.5 × 10^3^ bloodstream trypomastigotes. Total collagen fibers were detected by Masson’s Trichrome staining of cardiac tissue sections with collagen fibers stained blue. Bar = 50 µm. Percentage of cardiac fibrosis was quantified by ImageJ software version 1.50i (**E**) and are shown as the mean ± SE (n = 5). *p ≤ 0.05, one-way ANOVA *post hoc* Bonferroni tests of samples *vs*. vehicle.

The inflammatory mechanisms induced by infection of *T. cruzi*, combined with exposure to EtDAPs, lead to cardiac fiber destruction and myocarditis and induce an exacerbated initial reparative response (fibrosis) due to the cardiac remodeling associated with the development of CC, which is linked with HF and sudden death. To elucidate whether the increase in parasite burden and inflammation-related cell infiltration in the heart was associated with an increase in fibrosis during chronic ChD, we examined the histopathologic characteristics in the heart and observed a moderate formation of fibrotic *foci* in the heart tissue of animals infected with *T. cruzi* Ninoa 180 dpi. This fibrosis increased due to exposure to DEDTP (187%), DETP (107%) or DEP (72%) (Fig. 3) compared with the hearts of animals with high rates of infection (Supplementary Fig. S2H).

### Fibrotic damage was associated with changes in the proportion of total MΦ subpopulations

The induction of anti-inflammatory MΦ (M2) is particularly associated with fibrotic damage^34,35^ because they are an important source of profibrotic factors. Additionally, these cells acquire different phenotypes depending on the microenvironmental stimuli they receive, including those from toxicants. Our previous findings suggested that exposure to EtDAPs can modulate the phenotype and the responses of MΦ. Thus, exposure to EtDAPs could create an environment that promotes *T. cruzi* infection and tissue repair, which could lead to formation of fibrotic tissue.

To identify total MΦ populations in the PECs and phenotypically classify them into M1 or M2 types, we performed 7-color flow cytometry and sequential gating analyses (Supplementary Fig. S3) using the murine marker F4/80 and expression levels of MHC-II and TNF-α (M1), IL-10 and CD206 (M2a), MHC-II and CD206 (M2b), IL-10 and TGF-β (M2c), and IL-10 and VEGF (M2d).

**Figure 4:**
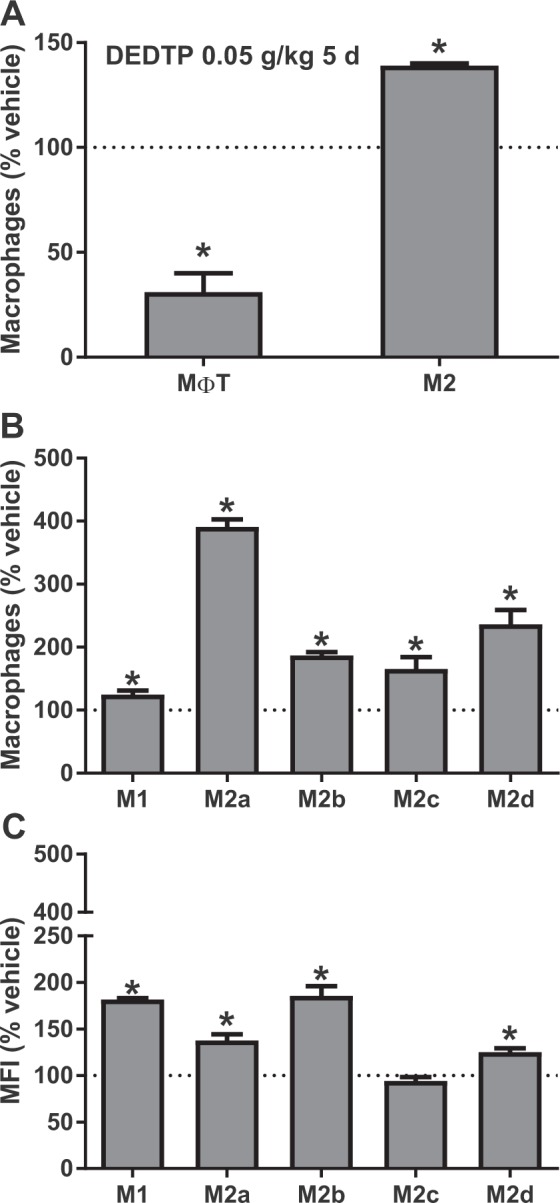
Early effect (5 days) of exposure to DEDTP on the proportion of macrophage subpopulations. PECs from female BALB/c mice administered with corn oil (vehicle group) or 0.05 g/kg body weight of DEDTP and infected (i.p.) with 7.5 × 10^3^ bloodstream trypomastigotes of *T. cruzi* after 24 h of exposure. Percentages of MΦT and alternative M2 macrophages (**A**). Percentages (**B**) and mean fluorescence index (MFI) (**C**) of classically activated/M1 or alternatively activated/M2a/b/c/d macrophages. Data represent the mean ± SE (n = 5). *p ≤ 0.05, Student’s t-test of samples *vs*. vehicle.

We quantified the MΦ in the PECs 5 days after female BALB/c mice were exposed to DEDTP. The results showed that the proportion of MΦT in the PECs decreased (69%), although we observed an increase (39%) in the proportion of the M2 subpopulation compared with that of the nonexposed animals (Fig. 4A). We further determined the subtypes of the MΦ phenotypes and observed that all the subtypes of MΦ (including M1) were increased. The proportion of increase in M1 was small (24%) (Fig. 4B) but was more important that of the M2 subtypes, where M2b and M2c increased 86 and 66%, respectively. Moreover, M2a (291%) and M2d (136%) showed a higher increase than found for M1 (Fig. 4B). In comparison, the mean fluorescence intensity (MFI), which is related to the activation status of the cells, indicated an increase in the activation of M1 (81%), M2a (38%), M2b (86%), and M2d (25%) (Fig. 4C), suggesting a unique correlation between the percentage of the cells and the proportion of the activated cells.

**Figure 5:**
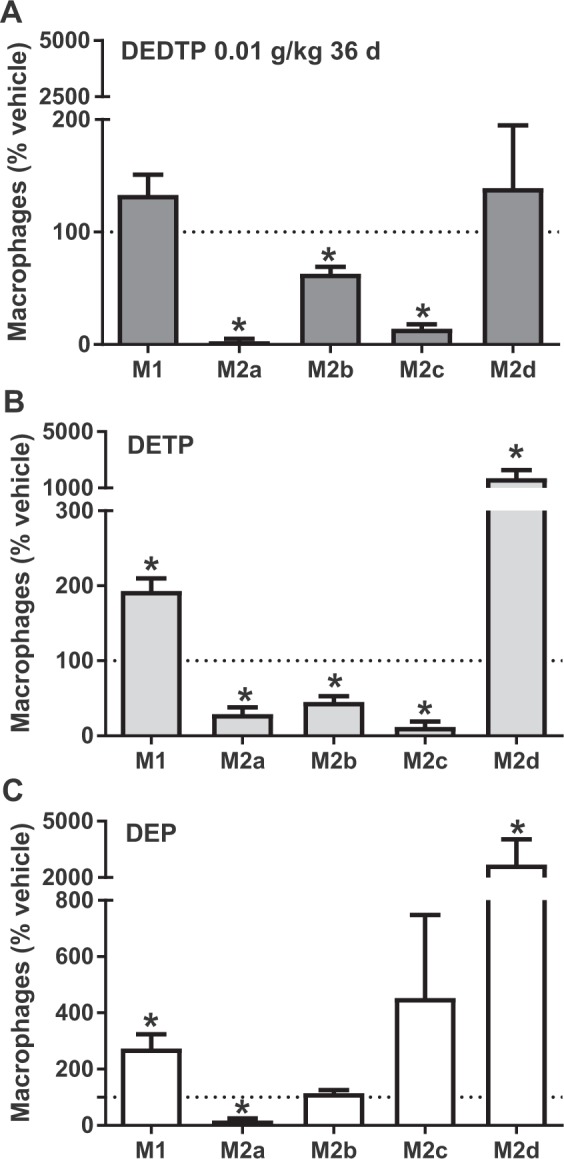
Long-term effect (36 days) of exposure to EtDAPs on the proportion of macrophage subpopulations. PECs from female C57BL/6J mice administered with corn oil (vehicle group) or 0.01 g/kg body weight of DEDTP (**A**), DETP (**B**) or DEP (**C**) and infected (i.p.) with 7.5 × 10^3^ bloodstream trypomastigotes of *T. cruzi* after 24 h of exposure. Percentage of classically activated/M1 and alternatively activated/M2a/b/c/d. Data represent the mean ± SE (n = 5). *p ≤ 0.05, Student’s t-test of samples *vs*. vehicle.

Finally, we evaluated the proportion of peritoneal MΦ subtypes in the C57BL/6J mice because we wanted to determine whether the exposure to EtDAPs could change the basally high proportion of the M1 macrophage phenotype in this strain into an M2 macrophage phenotype. The findings from flow cytometry analysis revealed no significant increase in the proportion of M1 and M2d cells, but the proportions of M2a, M2b and M2c MΦ in PECs of animals exposed to DEDTP were reduced (Fig. 5A). A similar trend was observed in the animals exposed to DETP, but the changes in all MΦ subtypes were significant (Fig. 5B), with a marked increase in the M2d subtype. The increase in the proportion of M1 and M2d was also evident in PECs from animals exposed to DEP (Fig. 5C). These data indicated that the basal immunological profile of each individual is important in the outcome after exposure to EtDAPs and that most EtDAPs induce a relevant increase in the M2d subtype of MΦ that is persistent over time and can be associated with the fibrotic damage observed in heart tissue.

## Discussion

Humans in many countries are constantly exposed to a variety of harmful contaminants and pathogens throughout their lifetimes that affect their health and reduce their lifespan^13^. Pesticides are ubiquitous contaminants that can enter the body by direct or indirect contact with contaminated food or by the use of products that contain contaminants as additives, resulting in chronical exposure that has been implicated in severe health adverse effects^9,36–38^. Some reports indicate that OP pesticides exert immunotoxic effects^39,40^; however, the effect of EtDAPs on the immune system is not fully understood. Recent studies revealed that metabolites derived from the degradation of OP compounds affect some cell types (lymphocytes, NK cells and MΦ) and mediators of the immune system (cytokines). These alterations can lead to an abnormal function of the immune response^19–21,41^. Moreover, the effects of exposure to EtDAPs are most likely to be underreported because EtDAPs are considered inert and safe^15^.

Infectious diseases are still an important cause of mortality and morbidity for millions of people around the world, and exposure to EtDAPs might play a role in interfering with their immune response against parasites. In this regard, a previous report using a mathematical model to describe the dynamics of within-host infection and the associated individual’s immune response showed that interactions between infection and pesticide exposure can promote the within-host parasite burden and decrease the host’s health status by reducing the immune response^6^. Therefore, we evaluated the effect of exposure to low doses of EtDAPs (0.01 g/kg), which had been previously administered i.p., on the development of an immune response against *T. cruzi* Ninoa infection *in vivo*. The dose given corresponds to low levels of chronic exposure in humans that are considered safe. In a report using a murine model exposed to OP compounds, the authors found that this type of exposure can act as an immunomodulator for MΦ, inducing them to express phenotypic characteristics of M2 cells. This particular subtype of macrophage is usually associated with anti-inflammatory responses that alter the response to an antigenic challenge^21^, and this dysregulation in macrophage polarization has clinical implications in chronic conditions, such as the pathogenesis of ChD.

*T. cruzi* is the etiologic agent of ChD and a potent stimulator of M1-type macrophage activation, with proinflammatory functions important for the control of infection during the acute phase that are thus involved in the outcome of the disease. Recently, studies showed that the infection can lead to progressive fibrotic inflammatory cardiomyopathy that results in permanent cardiac damage^42^. Due to the versatility and plasticity of MΦ to reprogram their phenotypes in a wide range of functional statuses (e.g., M1 to M2 as a simplistic example), depending on the environmental stimulus and physiological functions^43–45^, pollutants such as DEDTP with an immunomodulatory effect can exert adverse effects in individuals.

Our results demonstrate, for the first time, that a single exposure to a low level of DEDTP or DEP is enough to modify the development of an infection, such as with *T. cruzi*. We suggest that the elimination of parasites is reduced because MΦ are subsequently reprogrammed to switch to the immunosuppressive/tissue-repairing phenotype of M2 cells (mainly M2a and M2d), which contributes to uncontrolled parasite replication. These results are consistent with those observed in some human populations^17^, where chronic exposure to low levels of the OP pesticide chlorpyrifos exacerbated infection with *Leishmania sp*. and delayed the healing of cutaneous leishmaniasis lesions due to alterations in the immune response. This immunotoxicity could be associated with a significant increase in the level of the anti-inflammatory mediator arginase coupled with a significant decrease in the levels of the proinflammatory mediators NO and IFN-γ, as previously reported^18^.

On the other hand, exposure to DETP induced a novel behavior related to blood parasitemia. Exposure to DETP prior to parasitic infection reduced the parasite burden in the blood and delayed the resolution time of the acute phase, although the induction of the M2 cells and fibrosis in the heart after DETP exposure was similar to that caused by exposure to the other EtDAPs. It can be inferred that DETP could present a different and/or additional mechanism to induce immunosuppression, which may be attributed this difference. In this regard, we suggest that DETP could modify some immunological responses or activate other types of immune cells and therefore initiate a higher inflammatory response in the individual and a posterior induction of M2 cells, thus contributing to the formation of fibrotic tissue.

A number of studies have indicated that the heart is one of the principal target organs affected by *T. cruzi* infection because heart cells have a highly developed capacity for membrane repair that could increase their permissiveness to infection^46^. Cardiac tissue Infection may be accompanied by mononuclear cell infiltration that establishes an intense inflammatory condition that destroys the tissue^47^, displaces cardiac myofibers and activates reparative responses, thus producing fibrosis that decreases muscle contractibility and increases the possibility of congestive HF and host death.

Additionally, exposure to DEP produced pathological changes other than those of DEDTP or DETP because it caused a reduction in the weight of several organs, a condition usually related to the induction of systemic toxicity, such as in the case of exposure to the other OP pesticides^48,49^. DEP induced slight organ toxicity and reduced the mass of the heart, spleen and kidneys, suggesting an adverse long-term effect through the degradation of lipids and proteins^50,51^, which leads to organ atrophy, whereas similar effects were not found for DEDTP or DETP.

Our data improve our understanding of the role of OP metabolites as toxicity- mediating agents in the spread and aggressiveness of infectious diseases, which has been underestimated despite the significant human health risk. Therefore, new efforts must be made to carry out more research aimed at studying the impact of exposure to EtDAP metabolites on susceptible populations and to study the metabolic reactions associated with the degradation of EtDAPs that enable the establishment of different levels of individual susceptibility to minimize the economic impact of medical treatment costs incurred to treat the people affected worldwide and the effects on the quality of life of exposed individuals. Therefore, these findings support the call to regulate the use of EtDAPs in products for human consumption.

## Conclusions

The exposure to DAPs increases the parasite burden of an infection such as *T. cruzi* and induces the M2 polarization of macrophages, increasing fibrosis and inflammation in tissues such as that of the heart. Our study highlights an effect of EtDAPs on modulating the immune response to a protozoan infection, namely, *T. cruzi*. The exposure to EtDAPs, especially DEDTP, represents a health hazard in populations with a preexisting infection (e.g., with *T. cruzi*), as it could aggravate some conditions such as fibrosis in some organs. Direct or indirect exposure to EtDAPs should therefore be strictly monitored and controlled in occupationally exposed individuals as well as in the general population to determine their contribution to disabling diseases.

## Materials and Methods

### Materials

We obtained DEDTP and DETP from Sigma Aldrich, St. Louis, MO, USA (Cat. D93600-56; 90.0% purity and 445177, 98% purity, respectively), and DEP from Matrix Scientific, Columbia, SC, USA (Cat. 098586; 95% purity). The following antibodies were purchased from eBioscience Thermo Fisher Scientific (San Diego, CA, USA): anti-mouse F4/80 monoclonal antibody (BM8) eFluor 450 (Cat. 48-4801-82; 0.2 mg/ml), anti-mouse MHC Class II (I-A/I-E) monoclonal antibody (M5/114.15.2) APC-eFluor 780 (Cat. 47-5321-80; 0.2 mg/ml), anti-mouse CD309 (FLK1) monoclonal antibody (Avas12a1) APC (Cat. 17-5821-80; 0.2 mg/ml), anti-mouse latency-associated peptide (LAP) monoclonal antibody (TW7-16B4) PE-Cyanine7 (Cat. 25-9821-82; 0.2 mg/ml), anti-mouse TNF-α monoclonal antibody (MP6-XT22) with FITC (Cat. 11-7321-81; 0.5 mg/ml) and anti-mouse IL-10 monoclonal antibody (JES5-16E3) with PE (Cat. 12-7101-82; 0.2 mg/ml). BioLegend (San Diego, CA, USA) was the supplier of the anti-mouse CD206 (MMR) FITC monoclonal antibody (Cat. 141704; 0.5 mg/ml). We obtained the remaining reagents from AMRESCO, Inc. (Cochran Road Solon, OH, USA), J. T. Baker (Avantor, Phillipsburg, NJ, USA) and PISA Laboratories (Pachuca, Hgo, Mexico) as indicated.

### Ethics statement and animals

We obtained 6–10-week-old C57BL/6J and BALB/c mice from the Unit of Production and Care of Experimental Laboratory Animals (UPEAL) of the Center for Research and Advanced Studies of the National Polytechnic Institute (CINVESTAV). All procedures followed the Mexican Guideline Regulations of Animal Care and Maintenance (NOM-062-ZOO-1999, 2001) (https://www.gob.mx/senasica/documentos/nom-062-zoo-1999) and the Internal Ethics Committee for the Care and Use of Laboratory Animals (CICUAL). CICUAL-CINVESTAV approved the experimental protocol (Protocol 153-15); therefore, these animals were kept on a 12-h light/dark cycle at 20–22 °C and 40–60% humidity. The animals had access to food and water *ad libitum*. Animals were acclimated for 1 week prior to experimentation and were sacrificed through the administration of a 3% isoflurane overdose (PISA Laboratories).

### Experimental groups and treatment

The animals were randomly distributed into different groups (n = 5) that received intraperitoneal (i.p., 50 µl) administration of 0, 0.01 or 0.05 g/kg body weight of freshly prepared DEDTP, DETP or DEP or 50 µl of vehicle (corn oil or physiological saline). The lowest dose was selected based on a previous study that reported an apparent immunotoxicity in murine MΦ in mice exposed to DEDTP at 0.01 g/kg body weight^21^. The higher dose was selected based on the estimated levels observed in urine from the general population (healthy humans), rather than the levels from occupational exposure, to obtain physiological and environmentally relevant conditions^15,30,31^. The number of animals used in each experimental group was in accordance with the 3Rs principle (replacement, reduction and refinement) to obtain statistical significance.

### *In vivo* infection

All experiments were conducted using the trypomastigote phase of the Mexican *T. cruzi* Ninoa strain (MHOM/MX/1994/Ninoa), which was originally isolated from a patient in Oaxaca (Mexico)^52,53^; we selected this strain to develop an experimental ChD mouse model due to its low virulence and low mortality^54^.

We maintained the parasites by sequential murine passages. We obtained blood trypomastigotes at the peak of infection (15 days) from previously infected BALB/c mice, and we prepared aliquots with sterile phosphate-buffered saline solution (PBS) containing 7.5 × 10^3^ parasites in 100 µl. We injected i.p. an aliquot *per* BALB/c mouse to maintain the colony of parasites or to develop the ChD model^55^. The initial parasitemia was determined 8 days after the i.p. administered injection, and every four days 5 µl of blood was collected from the tail vein, and this blood sample (1:50 in physiological saline with 0.1% EDTA) was used to determine the number of parasites *per* milliliter using a hemocytometer and direct observation under with optical microscope.

### Histopathology

We collected the hearts of the animals 180 days postinfection (dpi), fixed them with 4% paraformaldehyde and embedded them in paraffin. We cut longitudinal 5 µm sections and stained them with hematoxylin and eosin (H&E) and Masson’s Trichrome following the standard protocols^56–58^. The inflammatory infiltrate and fibrotic lesions were evaluated in 20 consecutive fields using a light microscope. We used ImageJ software version 1.50i (available from NIH, Bethesda, MD, USA) to quantify the fibrotic surfaces in all samples.

### Organ weight

We obtained and weighed the heart, spleen, liver, lungs and kidneys of the animals after 180 dpi. We measured them in absolute values and transformed the values to relative weights: as organ (g) to body weight (g) multiplied by 100.

### Isolation of PECs

We obtained PECs by washing the peritoneal cavity of the mice with 8 ml ice-cold 1X PBS (NaCl 137 mM, KCl 2.7 mM, Na_2_HPO_4_ 10.1 mM, and KH_2_PO_4_ 1.8 mM; pH 7.4, J. T. Baker) plus 3% fetal bovine serum (FBS) injected i.p. After a soft massage of the peritoneum, we recovered the fluid and centrifuged the suspension at 453 × g for 5 min at 4 °C. We resuspended the cells at a density of 1 × 10^6^ cells/ml in ice-cold PBS.

### Flow cytometry analysis

We performed an analysis of the macrophage subpopulations in the PECs suspended in flow cytometry buffer (5% FBS, 0.02% sodium azide and 1X PBS) at a density of 1 × 10^6^ cells/ml. We stained them for extracellular markers with 1 µg/ml of anti-CD3, anti-CD4, anti-CD8 or anti-CD335; 0.3 µg/ml of anti-F4/80; 0.6 µg/ml anti-CD206; or 0.1 µg/ml of anti-MHC-II. We concomitantly stained the cells for intracellular contents with 0.1 µg/ml of anti-CD309 (VEGF) and anti-LAP (TGF-β), 0.3 µg/ml of anti-IL-10, and 0.6 µg/ml of anti-TNF-α antibodies by using fixation/permeabilization solution (Cytofix/Cytoperm, BD Biosciences, San Diego, CA, USA), following the manufacturer’s instructions. We quantified the stained cells (1 × 10^5^ events) using a LSRFortessa flow cytometer (BD Biosciences, San Diego, CA, USA), and we analyzed the data with FlowJo v10.0.7 software (Tree Star, Inc., Ashland, OR, USA). The data represent the percentage of positively labeled cells, as previously reported^20^. We identified total MΦ (MΦT) as F4/80^+^ cells and each cellular subpopulation as the percentage of cells expressing the markers MHC-II^+^TNF-α^+^ (M1), IL-10^+^CD206^+^ (M2a), MHC-II^+^CD206^+^ (M2b), IL-10^+^TGF-β^+^ (M2c) and IL-10^+^VEGF^+^ (M2d) from F4/80^+^ cells (Supplementary Fig. S3).

### Statistical analysis

All data were analyzed using GraphPad Prism 7.0 software (GraphPad Software, Inc., San Diego, CA, USA). Before using parametric tests, the assumption of normality was verified using the Kolmogorov-Smirnov test. A one-way ANOVA with a Bonferroni *post hoc* test or Student’s t-test was used, as appropriate, to identify differences between experimental groups. The data represent the mean ± standard error (SE), and a *p* value of less or equal to 0.05 (*p* ≤ 0.05) was considered statistically significant.

## Supplementary information

Supplementary Information

## Data Availability

The data that support the findings of this study are available from the corresponding author upon reasonable request.

## References

[1] Lopes MF, Freire-de-Lima CG, DosReis GA (2000). The macrophage haunted by cell ghosts: a pathogen grows. Immunol.Today.

[2] Hotez PJ (2007). Control of neglected tropical diseases. N. Engl. J. Med..

[3] Arnal A, Waleckx E, Rico-Chávez O, Herrera C, Dumonteil E (2019). Estimating the current burden of Chagas disease in Mexico: a systematic review and meta-analysis of epidemiological surveys from 2006 to 2017. PLoS Negl Trop Dis.

[4] Vieira JL (2019). Chagas cardiomyopathy in Latin America review. Curr Cardiol Rep.

[5] Echeverria LE, Morillo CA (2019). American Trypanosomiasis (Chagas Disease). Infect. Dis. Clin. North Am..

[6] Booton RD, Yamaguchi R, Marshall JA, Childs DZ, Iwasa Y (2018). Interactions between immunotoxicants and parasite stress: implications for host health. J. Theor. Biol..

[7] Reemtsma T, Quintana JB, Rodil R, García M, Rodríguez I (2008). Organophosphorus flame retardants and plasticizers in water and air I. occurrence and fate. Trends Analyt Chem..

[8] Terry A (2012). Functional consequences of repeated organophosphate exposure: potential non-cholinergic mechanisms. Pharmacol. Ther..

[9] Chawla P, Kaushik R, Swaraj VS, Kumar N (2018). Organophosphorus pesticides residues in food and their colorimetric detection. Environ Nanotechnol Monit Manag.

[10] Roberts, J. R. & Reigart, J. R. *Recognition and Management of Pesticide Poisonings*.https://www.epa.gov/sites/production/files/201501/documents/rmpp_6thed_final_lowresopt.pdf (2013).

[11] Sidhu GK (2019). Toxicity, monitoring and biodegradation of organophosphate pesticides: a review. Crit Rev Environ Sci Technol..

[12] Mennillo E, Cappelli F, Arukwe A (2019). Biotransformation and oxidative stress responses in rat hepatic cell-line (H4IIE) exposed to organophosphate esters (OPEs). Toxicol. Appl. Pharmacol..

[13] Landrigan PJ (2018). The Lancet commission on pollution and health. Lancet.

[14] Sudakin DL, Stone DL (2011). Dialkyl phosphates as biomarkers of organophosphates: the current divide between epidemiology and clinical toxicology. Clin. Toxicol..

[15] Kavvalakis MP, Tsatsakis AM (2012). The atlas of dialkylphosphates; assessment of cumulative human organophosphorus pesticides’ exposure. Forensic Sci. Int..

[16] Ezzat S (2005). Associations of pesticides, HCV, HBV, and hepatocellular carcinoma in Egypt. Int J Hyg Environ Health.

[17] Al-Dawood AN, Al-Ghazal RA, Al-Jaser MH, Khalil GM (2009). Effect of chlorpyrifos on healing of cutaneous leishmaniasis lesions after treatment with Pentostam®. Saudi J Biol Sci.

[18] Ashour M, Mohamed M, Elsawy B (2011). Adverse effects of organophosphorus insecticides on macrophage activity in persons at high risk for parasitic infection. Open Access Maced J Med Sci.

[19] Esquivel-Sentíes M, Vega L (2012). Organophosphorous pesticides metabolite reduces human T CD8 homeostasis and proliferation by inducing cellular death. J Environ Anal Toxicol.

[20] Esquivel-Sentíes M, Barrera I, Ortega A, Vega L (2010). Organophosphorous pesticide metabolite (DEDTP) induces changes in the activation status of human lymphocytes by modulating the interleukin 2 receptor signal transduction pathway. Toxicol. Appl. Pharmacol..

[21] Medina-Buelvas D, Estrada-Muñiz E, Flores-Valadez M, Vega L (2019). Genotoxic and immunotoxic effects of the organophosphate metabolite diethyldithiophosphate (DEDTP) *in Vivo*. Toxicol. Appl. Pharmacol..

[22] Burkholder B (2014). Tumor-induced perturbations of cytokines and immune cell networks. Biochim Biophys Acta Rev Cancer.

[23] Epelman S, Lavine KJ, Randolph GJ (2014). Origin and functions of tissue macrophages. Immunity.

[24] Murray PJ, Wynn TA (2011). Protective and pathogenic functions of macrophage subsets. Nat. Rev. Immunol..

[25] Rodgers KE, Imamura T, Devens BH (1985). Investigations into the mechanism of immunosuppression caused by acute treatment with O, O, Sttrimethyl phosphorothioate. I. Characterization of the immune cell population affected. Immunopharmacology.

[26] Rodgers KE, Imamura T, Devens BH (1987). Investigations into the mechanism of immunosuppression caused by acute treatment with O, O, S-trimethyl phosphorothioate: generation of suppressive macrophages from treated animals. Toxicol. Appl. Pharmacol..

[27] Rodgers K, Ellefson D (1988). Effects of acute administration of O, O, S-trimethyl phosphorothioate on the respiratory burst and phagocytic activity of splenic and peritoneal leukocytes. Agents and actions.

[28] Rodgers K, Ellefson D (1990). Modulation of macrophage protease activity by acute administration of O, O, S trimethyl phosphorothioate. Agents and actions.

[29] Pakshir P, Hinz B (2018). The big five in fibrosis: macrophages, myofibroblasts, matrix, mechanics, and miscommunication. Matrix Biol..

[30] Bouchard MF, Bellinger DC, Wright RO, Weisskopf MG (2010). Attention-deficit/hyperactivity disorder and urinary metabolites of organophosphate pesticides. Pediatrics.

[31] Bradman A (2005). Organophosphate urinary metabolite levels during pregnancy and after delivery in women living in an agricultural community. Environ. Health Perspect..

[32] Bryan MA, Guyach SE, Norris KA (2010). Specific humoral immunity versus polyclonal B cell activation in Trypanosoma cruzi infection of susceptible and resistant mice. PLoS Negl Trop Dis.

[33] Hoft DF, Lynch RG, Kirchhoff LV (1993). Kinetic analysis of antigen-specific immune responses in resistant and susceptible mice during infection with Trypanosoma cruzi. J. Immunol..

[34] Tang PM, Nikolic-Paterson DJ, Lan H-Y (2019). Macrophages: versatile players in renal inflammation and fibrosis. Nat Rev Nephrol..

[35] Braga TT, Agudelo JS, Camara NO (2015). Macrophages during the fibrotic process: M2 as friend and foe. Front Immunol.

[36] Möller A (2012). Organophosphorus flame retardants and plasticizers in airborne particles over the Northern Pacific and Indian Ocean toward the polar regions: evidence for global occurrence. Environ. Sci. Technol..

[37] Todd, G. D. *et al*. *Toxicological Profile for Phosphate Ester Flame Retardants*, https://www.atsdr.cdc.gov/toxprofiles/tp202.pdf (2012). 37262202

[38] Saillenfait AM, Ndaw S, Robert A, Sabaté JP (2018). Recent biomonitoring reports on phosphate ester flame retardants: a short review. Arch. Toxicol..

[39] Corsini E, Sokooti M, Galli C, Moretto A, Colosio C (2013). Pesticide induced immunotoxicity in humans: a comprehensive review of the existing evidence. Toxicology.

[40] Li Q (2007). New mechanism of organophosphorus pesticide-induced immunotoxicity. J Nippon Med Sch.

[41] Lima A, Vega L (2005). Methyl-parathion and organophosphorous pesticide metabolites modify the activation status and interleukin-2 secretion of human peripheral blood mononuclear cells. Toxicol. Lett..

[42] Bonney KM, Engman DM (2008). Chagas heart disease pathogenesis: one mechanism or many?. Curr. Mol. Med..

[43] Mantovani A (2004). The chemokine system in diverse forms of macrophage activation and polarization. Trends Immunol..

[44] Sica A, Mantovani A (2012). Macrophage plasticity and polarization: *in vivo* veritas. J. Clin. Invest..

[45] Martinez FO, Gordon S (2014). The M1 and M2 paradigm of macrophage activation: time for reassessment. F1000Prime Rep.

[46] Fernandes MC, Andrews NW (2012). Host cell invasion by Trypanosoma cruzi: a unique strategy that promotes persistence. FEMS Microbiol. Rev..

[47] Gutierrez F, Guedes P, Gazzinelli R, Silva J (2009). The role of parasite persistence in pathogenesis of Chagas heart disease. Parasite Immunol..

[48] Aroonvilairat S (2018). Effects of topical exposure to a mixture of chlorpyrifos, cypermethrin and captan on the hematological and immunological systems in male Wistar rats. Environ. Toxicol. Pharmacol..

[49] Mansour SA, Mossa AT (2010). Oxidative damage, biochemical and histopathological alterations in rats exposed to chlorpyrifos and the antioxidant role of zinc. Pestic Biochem Physiol.

[50] Mossa AT, Refaie AA, Ramadan A (2011). Effect of exposure to mixture of four organophosphate insecticides. Res. J. Environ. Toxicol..

[51] Mansour SA, Mossa A-TH (2011). Adverse effects of exposure to low doses of chlorpyrifos in lactating rats. Toxicol Ind Health.

[52] Bosseno MF (2002). Predominance of Trypanosoma cruzi lineage I in Mexico. J. Clin. Microbiol..

[53] Monteón VM (1996). American trypanosomosis: *in situ* and generalized features of parasitism and inflammation kinetics in a murine model. Exp. Parasitol..

[54] Espinoza B (2010). Mexican Trypanosoma cruzi (TCI) strains with different degrees of virulence induce diverse humoral and cellular immune responses in a murine experimental infection model. J. Biomed. Biotechnol..

[55] Terrazas CA (2011). MIF synergizes with Trypanosoma cruzi antigens to promote efficient dendritic cell maturation and IL-12 production via p38 MAPK. Int. J. Biol. Sci..

[56] Pérez-Vargas JE (2014). Hesperidin prevents liver fibrosis in rats by decreasing the expression of nuclear factor-κB, transforming growth factor-β and connective tissue growth factor. Pharmacology.

[57] Hernández-Aquino E (2017). Naringenin prevents experimental liver fibrosis by blocking TGFβ-Smad3 and JNK-Smad3 pathways. World J. Gastroenterol..

[58] Casas‐Grajales S (2017). Quercetin reverses experimental cirrhosis by immunomodulation of the proinflammatory and profibrotic processes. Fundam Clin Pharmacol.

