# Supplementary material for: Increased heart fibrosis and acute infection in a murine Chagas disease model associated with organophosphorus pesticide metabolite exposure
Source: Sci Rep. 2019 Nov 26;9:17539. doi: 10.1038/s41598-019-54218-7 (PMC6879754; doi:10.1038/s41598-019-54218-7)
Supplement: Supplementary file 1 — Supplementary Information [file 41598_2019_54218_MOESM1_ESM.pdf]

Supplementary Material

Increased heart fibrosis and acute infection in a murine Chagas disease model associated with organophosphorus pesticide metabolite exposure

Dunia Margarita Medina-Buelvas[1], Elizabet Estrada Muñiz[1], Miriam Rodríguez-Sosa[2], Mineko Shibayama[3] and Libia Vega[1,*]

[1]Department of Toxicology, Centro de Investigación y de Estudios Avanzados del Instituto Politécnico Nacional. Av. Instituto Politécnico Nacional 2508, San Pedro Zacatenco, CP 07360, Gustavo A. Madero, Ciudad de México, México; dmedinab@cinvestav.mx (D.M.-B.); eestrada@cinvestav.mx (E.E.-M.).

[2]Biomedicine Unit, Facultad de Estudios Superiores Iztacala, Universidad Nacional Autónoma de México (UNAM). Avenida de los Barrios 1, Los Reyes Iztacala, CP 54090 Tlalnepantla, Estado de México, México; rodriguezm@campus.iztacala.unam.mx.

[3]Department of Infectomics and Molecular Pathogenesis, Centro de Investigación y de Estudios Avanzados del Instituto Politécnico Nacional. Av. Instituto Politécnico Nacional 2508, San Pedro Zacatenco, CP 07360, Gustavo A. Madero, Ciudad de México, México; mineko@cinvestav.mx

*Corresponding author

Department of Toxicology, Centro de Investigación y de Estudios Avanzados del Instituto Politécnico Nacional. Av. Instituto Politécnico Nacional 2508, San Pedro Zacatenco, CP 07360, Gustavo A. Madero, Ciudad de México, México. E-mail: lvega@cinvestav.mx. Phone: 01 55 5747 3800. Fax: 01 55 5747 7002.

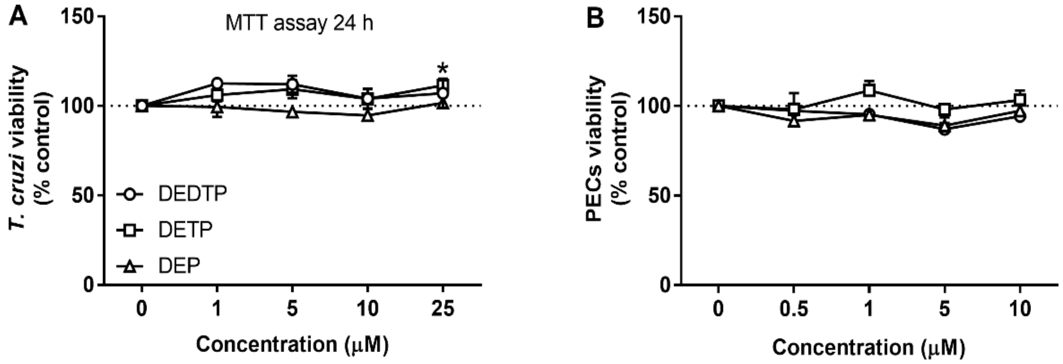

Figure S1. Cell viability of *T. cruzi* parasites and macrophages exposed to different concentrations of EtDAPs determined by the MTT assay. *T. cruzi* parasites were exposed to 1-25 µM of EtDAPs for 24 h (**A**) and PECs were exposed to 0.5-10 µM EtDAPs for 24 h (**B**). Data represent the mean ± SE (n = 3) as the percentage of the vehicle (100%). *$p \leq 0.05$, one-way ANOVA *post hoc* Bonferroni tests of samples *vs.* vehicle.

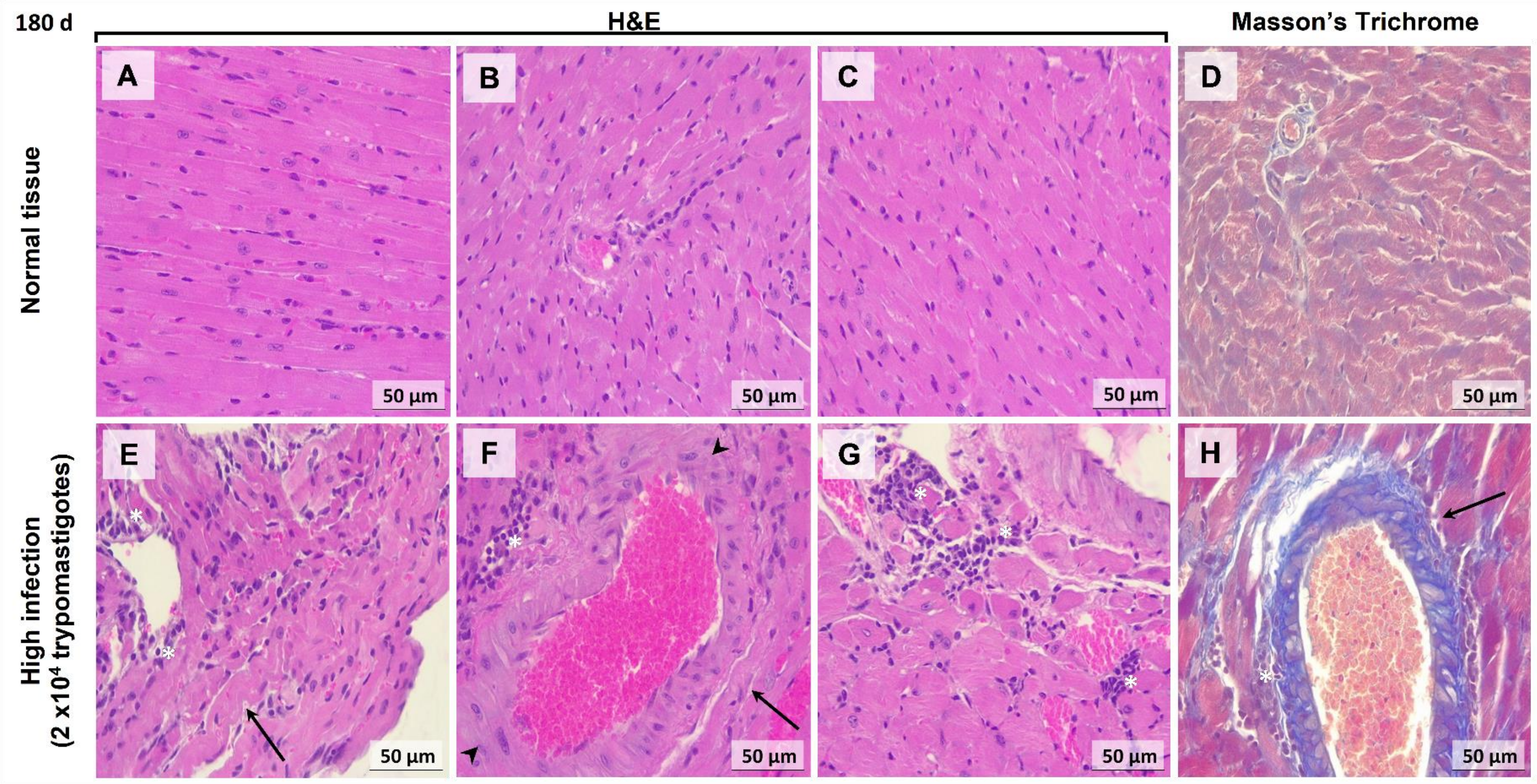

Figure S2. Representative images from H&E and Masson's Trichrome staining of heart sections (5 µm) from normal and highly infected female BALB/c mice (2 x 10^4 bloodstream *T. cruzi* trypomastigotes) after 180 days. Staining of normal mature heart showed a homogeneous and intact cardiac structure (**A**-**D**) and the heart sections of highly infected mice showed fiber destruction (black arrows), collagen deposition (closed arrowhead) and inflammatory reaction (white asterisk) and fibrosis stained in blue. Bar = 50 µm.

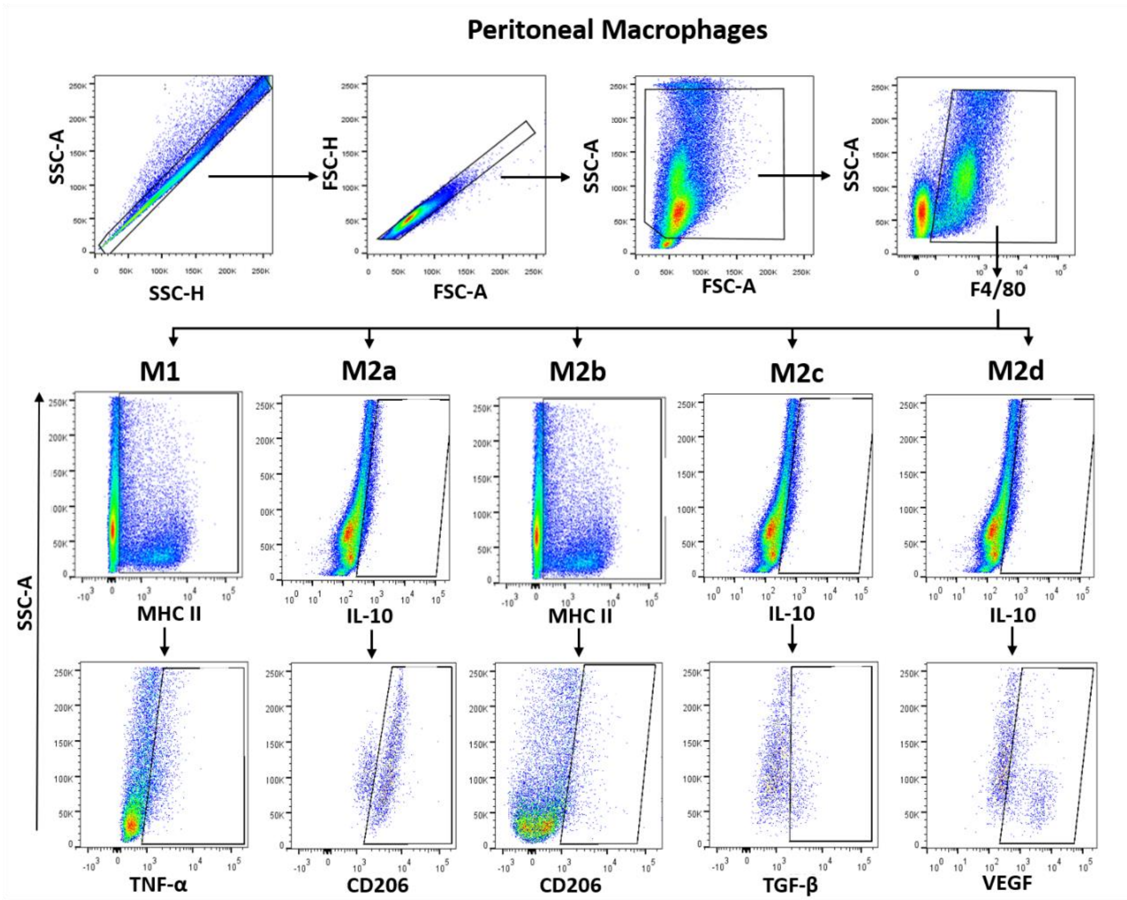

Figure S3. Representative flow cytometry dot plots showing the gating strategy for the phenotypic characterization of macrophages. Single cells were identified by comparing side scatter-area (SSC-A) to side scatter-height (SSC-H) and forward scatter-area (FSC-A) to forward scatter-height (FSC-H), the macrophage population was discriminate by morphology cell size (FSC-A) and granularity (SSC-A) and the MΦT macrophages were identified by F4/80 expression and SSC characteristics. Macrophages were phenotypificated based on the expression of MHC-II and TNF-α (M1), IL-10 and CD206 (M2a), MHC-II and CD206 (M2b), IL-10 and TGF-β (M2c), or IL-10 and VEGF (M2d).
